# Patient involvement in medical decision-making and pain among elders: physician or patient-driven?

**DOI:** 10.1186/1472-6963-5-4

**Published:** 2005-01-14

**Authors:** Tyrone F Borders, Ke Tom Xu, James Heavner, Gina Kruse

**Affiliations:** 1 Department of Health Management and Policy, University of North Texas School of Public Health, 3500 Camp Bowie Blvd., Fort Worth, TX 76107, USA; 2 Division of Health Services Research, Texas Tech University School of Medicine, Lubbock, Texas, USA; 3 Department of Anesthesiology, Texas Tech University School of Medicine, Lubbock, Texas, USA; 4 Baylor Medical School, Houston, Texas, USA

## Abstract

**Background:**

Pain is highly prevalent among older adults, but little is known about how patient involvement in medical decision-making may play a role in limiting its occurrence or severity. The purpose of this study was to evaluate whether physician-driven and patient-driven participation in decision-making were associated with the odds of frequent and severe pain.

**Methods:**

A cross-sectional population-based survey of 3,135 persons age 65 and older was conducted in the 108-county region comprising West Texas. The survey included self-reports of frequent pain and, among those with frequent pain, the severity of pain.

**Results:**

Findings from multivariate logistic regression analyses showed that higher patient-driven participation in decision-making was associated with lower odds (OR, 0.82; 95% CI, 0.75–0.89) of frequent pain, but was not significantly associated with severe pain. Physician-driven participation was not significantly associated with frequent or severe pain.

**Conclusions:**

The findings suggest that patients may need to initiate involvement in medical decision-making to reduce their chances of experiencing frequent pain. Changes to other modifiable health care characteristics, including access to a personal doctor and health insurance coverage, may be more conducive to limiting the risk of severe pain.

## Background

Persons age 65 years and older commonly endure a multitude of chronic and debilitating conditions which contribute to persistent pain [1]. Estimates of the prevalence of pain among the community-dwelling elderly range between 25% and 50% [2,3]. Pain has been found to have a substantial effect on health-related quality of life [2], the use of over the counter and prescription drugs [1,4], and the utilization of medical care [5]. As the number of elderly persons in the United States rises, more research is needed to determine how the delivery of medical care could be altered to limit the onset of pain and its subsequent burden on health status and the health care system.

Increasing patients' involvement in the medical decision-making process is one potentially fruitful means of improving pain management. Several studies suggest that patients, especially those with chronic conditions, who have opportunities to participate in care have more positive health outcomes than those who do not [6,7]. While other studies have pointed out that the positive correlation between patient participation and health outcomes is more suggestive than conclusive, Guadagnoli and Ward have stated that physicians should nevertheless strive to engage their patients in decision-making for humanitarian reasons [8].

Although patients' participation may improve their health outcomes, the effect can be diminished among elderly patients. Elderly patients, as compared to younger patients, have been shown to be less participatory in medical-decision making [9-11]. Using a longitudinal cohort, the Medical Outcomes Study found that patients older than 75 years were less participatory [12]. Other studies have also shown that older people tend to exhibit more conversational behaviors [13], give more socially desirable responses [14], and defer to physicians' authorities [15].

The primary objective of the present study was to examine how participation in decision-making was associated with the occurrence of pain among a cohort of community-dwelling elders. In contrast to previous studies, we differentiated two types of participation in decision-making. The first type is physician-driven in which the physician takes the initiative to ask questions and offer choices to patients. The second type is patient-driven, in which the patient takes the initiative to ask questions and express preferences. We hypothesized that stronger physician and patient-driven participation in decision-making would be associated with lower odds of frequent pain and, among those with frequent pain, lower odds of severe pain. We also tested for the effects of other health care factors which might be conducive to pain management, such as tenure with a personal doctor. The findings have implications for how older patients interact with their physicians as well as how physicians and clinic managers organize health services.

## Methods

### Sample and setting

Data were obtained through a longitudinal, population-based study of community-dwelling elders, the Texas Tech 5000 Survey. The Texas Tech 5000 Survey was conducted in a 108-county region of West Texas, a geographically and ethnically diverse area encompassing approximately half of the state's land mass. The survey has been described in detail elsewhere [16-21]. Briefly, approximately 65,000 households were randomly selected from residential telephone listings and screened to identify a cohort of 5,000 individuals age 65 years and older. Age-qualified individuals were subsequently tested for cognitive impairment using a telephone version of the Mini Mental State Evaluation [22]. Ninety-three percent of individuals did not have impairment and thus were eligible for participation in the study. Excluding telephone numbers that were never reached, those who refused the cognitive screener, and individuals who failed the cognitive screener, the eligible sample size was 6,942. Two follow-up surveys have since been conducted among the original cohort. Selected questions measuring satisfaction with care and health-related quality of life were included in each wave. To limit respondent burden, most questions were asked only during one wave (the patient and physician-driven participation in decision-making and perceived pain questions were only asked during Wave 3). Of the 6,942 households that were eligible for participation in Wave 1 of the survey, 5,006 persons participated, yielding a baseline response rate of 72%. The data presented here are from 3,135 subjects who participated in all three waves of the survey, yielding an overall response rate of approximately 45%. While some subjects were obviously lost to follow-up, the demographic composition of the study samples remained similar over the study period. The Texas Tech Health Sciences Center Institutional Review Board for the Protection of Human Subjects approved the study.

### Measures

#### Frequent and severe pain

The occurrence and severity of pain were measured in Wave 3 using items developed for and included in two nationally representative surveys of older persons, the Health and Retirement Study (HRS) and Assets and Health Dynamics among the Oldest Old (AHEAD) [23]. First, the frequency of pain was measured by asking respondents if they were "often troubled with pain?" Responses were categorized to distinguish those persons who were often troubled (referred to hereafter as frequent pain) and those who were not often troubled by pain, as has been done in previous studies [2]. The severity of pain was assessed among those persons who reported frequent pain through a single item asking, "how bad is the pain most of the time?" Responses were categorized to differentiate those persons with mild or moderate pain versus severe pain.

#### Sociodemographic factors

A number of sociodemographic, health care, and health status measures were included. Sociodemographic factors were gender, age (continuous), marital status, educational status (high school graduate vs. less than high school education), and place of residence (urban, rural, and frontier). An urban area is a metropolitan county, or a county with a total population of at least 50,000, whereas a rural area is a county with fewer than 50,000 persons. Rural counties were further classified according to whether they were frontier areas, or counties with fewer than 7 persons per square mile [24].

#### Health care factors

Health care variables were health insurance coverage, the number of physician visits in the last 6 months, tenure with a personal doctor, an index measuring physician-driven participation in decision-making, and an index measuring patient-driven participation in decision-making. Health insurance coverage was coded as Medicaid, Medicare, Medicare plus other private or government coverage, other private or government coverage, and no insurance. Tenure with a personal doctor was measured using a single question asking if the individual had a personal doctor and, if so, the duration of tenure with the physician (less than 1 year, 1 to 2 years, 3 to 4 years, and 5 or more years). An index of physician-driven participation in decision-making was created using three items taken from the Medical Outcomes Study [12]. The physician-driven participation in decision-making questions included: 1) How often does your doctor ask you to help make the decision when there is a choice between treatments?, 2) How often does your doctor give you some control over your treatment?, and 3) How often does your doctor ask you to take some of the responsibility? Response options for each item ranged from 0 (never) to 4 (very often). The aggregation of the three items divided by the total number of items produces a score between 0 and 4 with a higher index score indicating greater involvement. For the present data set, the physician-driven participation index had reasonable internal consistency (Cronbach's alpha = 0.69), which was similar to that found in the Medical Care Outcomes Study (Cronbach's alpha = 0.74) [12].

Three questions adopted from a study of older patients' communication during medical visits [25] were used to measure patient-driven participation in decision-making. These questions included: 1) How often do you write out a list of symptoms, complaints, and medications before visiting a doctor?, 2) How often do you express preferences for tests, medications, and treatments?, and 3) How often do you call to clarify information or report symptoms or side effects after a visit? As was the case for the physician-driven index, the patient-driven participation index ranges between 0 and 4 with a higher score indicating greater involvement. The Cronbach's alpha for the patient-driven participation index was 0.58.

#### Health status

Overall health status (categorized as excellent, very good, good, and fair or poor) was measured using a general health item from a brief health-related quality of life instrument (the SF-12) [26]. Mental health status was assessed with the mental component score (MCS) of the SF-12. Additional health status variables included whether the individual had ever been diagnosed with arthritis and the number of additional comorbid conditions (categorized as none, one, two, and three or more).

### Statistical analysis

Chi-square tests were first conducted to determine whether there was an association between each categorical sociodemographic, health care, and health status factor and frequent and severe pain. T-tests were conducted to determine if there was a difference in each continuous sociodemographic, health care, and health status factor between individuals with and without frequent pain and individuals with and without severe pain. Next, multivariate logistic regression analyses were conducted to determine if physician or patient-driven participation in decision-making were associated with the odds of frequent pain and, among those with frequent pain, the odds of severe pain. The potential for multicollinearity between the covariates was assessed by calculating their variance inflation factors; no problems with multicollinearity were found.

## Results

### Description statistics for individuals with frequent pain

A total of 1,333 (42.5%) of the 3,135 survey participants had frequent pain. Table 1 presents percentages for frequent pain by categorical sociodemographic, health care, and health status variables. Several categorical sociodemographic variables were significantly associated with frequent pain. Frequent pain was less common among males (compared to females) and among married persons (compared to single persons). Frequent pain was more common among persons with less than a high school degree as compared to those with at least a high school degree. Health insurance was insignificant, but tenure with a personal doctor was associated with frequent pain. Specifically, pain was least common among individuals with no personal doctor, compared to those who had a personal doctor. Frequent pain was more common among persons with more comorbid diseases or conditions (compared to those with none), those with arthritis (compared to those without arthritis), and those with poorer self-rated general health. As shown in Table 2, persons with frequent pain had a higher number of physician visits in the previous six months but lower physician and patient-driven participation than those without frequent pain. Moreover, those with frequent pain had worse (lower) mental component scores (MCS) than those without frequent pain.

**Table 1:** Prevalence of frequent and severe pain by categorical independent variables

Sociodemographics		N = 3,135	Frequent Pain %	Severe Pain %
Gender	Male	952	35.7^1^	6.3^3^
	Female	2,183	45.5	10.4
Race/Ethnicity	White	2,678	42.5	8.4^1^
	Hispanic	327	42.5	11.6
	Other	130	43.9	19.2
Education	< high sch. grad.	785	45.9^3^	13.3^1^
	High school grad.	2,350	41.4	7.8
Marital Status	Married	1,661	40.8^3^	8.1
	Not married	1,474	44.5	10.3
Rural / urban residency	Urban	1,720	43.9	9.6
	Rural, non-frontier	1,072	40.8	8.7
	Frontier	343	41.1	8.5
Income	<$10,000	491	47.9^1^	14.3^2^
	$10–20,000	658	47.7	10.3
	$21–30,000	505	43.0	7.7
	$31,000 and higher	892	36.0	5.7
Health care				
Insurance	Uninsured	93	46.2	21.5^1^
	Medicare	917	41.8	9.2
	Medicaid	341	43.7	12.6
	Medicare+other ins.	1,498	43.4	7.9
	Other private/gov. ins	286	37.8	7.7
Tenure with doctor	No personal doctor	426	37.8^1^	10.6
	Less than 1 yr	227	44.9	9.2
	1–2 yrs	414	45.7	10.1
	3–4 yrs	454	42.3	8.4
	5 or more yrs	1,614	42.7	8.7
Health status				
No. of comorbidities	0	1,690	37.2^3^	6.3^2^
	1	874	47.1	10.5
	2	384	48.7	14.1
	3 or more	187	56.7	18.7
Ever diagnosed with arthritis	Yes	1,988	55.0^1^	12.8^2^
	No	1,147	20.8	2.9
General health status	Excellent	316	15.2^3^	2.2^3^
	Very good	720	31.1	5.6
	Good	1,040	41.0	3.1
	Fair	687	55.8	13.8
	Poor	360	69.2	29.2

^1 ^p < 0.0001, ^2 ^p < 0.01, ^3 ^p < 0.05

note: Analyses of severe pain were limited to those with frequent pain.

**Table 2:** Means and standard deviations for continuous independent variablesby frequent and severe pain

	Frequent pain		Severe pain	
	Yes	No	Yes	No
Sociodemographics				
Mean age (SD)	75.4(6.3)	75.3(6.3)	75.7(6.7)	75.3(6.3)
Health care				
Mean no. physician visits (SD)	6.3(8.5)^1^	3.9(6.1)	8.0(9.5)^1^	5.8(8.1)
Mean physician-driven participation index (SD)	1.8(1.2)^3^	1.9(1.2)	1.9(1.2)^3^	1.7(1.2)
Mean patient-driven participation index (SD)	2.4(1.0)^1^	2.7(1.0)	2.4(1.0)	2.4(1.1)
Health status				
Mean SF-12 mental component score (SD)	52.5(9.9)^1^	54.8(7.4)	48.7(12.1)^1^	53.6(9.0)

^1 ^p < 0.0001, ^2 ^p < 0.01, ^3 ^p < 0.05

note: Analyses of severe pain were limited to those with frequent pain.

### Description statistics for individuals with severe pain

Among the 1,333 individuals with frequent pain, a total of 287 (21.5%) had severe pain. As shown in Table 1, severe pain was less common among males (compared to females) but more common among other races/ethnicities (compared to non-Hispanic whites), persons with less than a high school degree (compared to at least a high school degree), and persons with lower household income. Severe pain was most common among individuals without health insurance. It was more common among persons with more comorbid diseases or conditions (compared to those with none), those with arthritis (compared to those without arthritis), and those with poorer self rated general health status. As shown in Table 2, those with severe pain had more physician visits and higher physician participation in decision-making. Those with severe pain had lower or worse mental component scores and a higher number of physician visits in the previous six months (compared to those without severe pain).

### Multivariate analyses of the odds of frequent pain

Findings from multivariate logistics analyses are shown in Table 3. Males had lower odds (OR, 0.81; 95% CI 0.67, 0.98) of frequent pain than females. Race/ethnicity was not significantly associated with frequent pain. Compared to urban residents, those residing in a rural area had lower odds (OR, 0.77; 95% CI 0.64, 0.92) of frequent pain than urban residents. Income, marital status, and frontier residence were not significantly associated with the odds of frequent pain. Individuals who had more physician visits in the previous six months had a higher odds of frequent pain (OR, 1.02; 95% CI 1.01, 1.04).

**Table 3:** Multivariate logistic regression of sociodemographic, health care, and health status factors on frequent and severe pain

Variable (reference group)	Frequent pain OR (95% CI)	Severe pain OR (95% CI)
Sociodemographics		
Age	0.99 (0.97, 1.00)	1.00 (0.98, 1.02)
Male (vs. female)	0.81 (0.67, 0.98)^3^	0.78 (0.54, 1.14)
Race/ethnicity		
Hispanic (vs. non-Hispanic white)	0.74 (0.54, 1.01)	0.74 (0.44, 1.26)
Other (vs. non-Hispanic white)	0.84 (0.56, 1.26)	2.28 (1.24, 4.21)^2^
Less than high school grad. (vs. grad.)	0.97 (0.78, 1.20)	1.08 (0.76, 1.55)
Married (vs. single)	0.92 (0.77, 1.10)	1.00 (0.72, 1.39)
Residence		
Rural (vs. urban)	0.77 (0.64, 0.92)^3^	0.89 (0.65, 1.23)
Frontier (vs. urban)	0.86 (0.66, 1.12)	0.78 (0.48, 1.28)
Income		
$10–20,000 (vs. < $10,000)	1.06 (0.81, 1.39)	0.81 (0.52, 1.26)
$21–30,000 (vs. < $10,000)	1.02 (0.75, 1.38)	0.96 (0.57, 1.62)
$31,000 and higher (vs. < $10,000)	0.90 (0.67, 1.20)	0.87 (0.52, 1.46)
Missing (vs. < $10,000)	0.95 (0.72, 1.25)	0.99 (0.63, 1.56)
		
Health care		
Insurance		
Medicare (vs. uninsured)	0.69 (0.41, 1.16)	0.41 (0.19, 0.86)^3^
Medicaid (vs. uninsured)	0.72 (0.44, 1.18)	0.35 (0.17, 0.71)^2^
Medicare plus other. (vs. uninsured)	0.85 (0.52, 1.39)	0.31 (0.15, 0.64)^2^
Other private/gov. ins. (vs. uninsured)	0.64 (0.37, 1.09)	0.34 (0.15, 0.76)^2^
No. of physician visits past 6 months	1.02 (1.01, 1.04)^2^	1.02 (1.00, 1.03)
Physician-driven participation index	0.99 (0.91, 1.06)	1.14 (0.99, 1.30)
Patient-driven participation index	0.82 (0.75, 0.89)^3^	0.93 (0.80, 1.09)
Tenure with doctor		
Less than 1 year (vs. no personal doctor)	0.92 (0.64, 1.33)	0.51 (0.27, 0.98)^3^
1–2 years (vs. no personal doctor)	0.95 (0.70, 1.30)	0.74 (0.43, 1.25)
3–4 years (vs. no personal doctor)	0.87 (0.64, 1.18)	0.56 (0.33, 0.96)^3^
5 or more years (vs. no personal doctor)	0.92 (0.71, 1.19)	0.65 (0.42, 0.99)^3^
		
Health status		
No. of comorbidities		
1 (vs. none)	1.06 (0.87, 1.27)	1.18 (0.84, 1.66)
2 (vs. none)	0.96 (0.74, 1.25)	1.50 (0.99, 2.28)
3 or more (vs. none)	1.01 (0.71, 1.43)	1.51 (0.90, 2.50)
Ever diagnosed with arthritis (vs. never)	3.62 (3.03, 4.33)^1^	1.54 (1.01, 2.35)^3^
SF-12 mental component score	0.99 (0.98, 1.00)	0.98 (0.97, 1.00)
General health status		
Excellent (vs. poor)	0.14 (0.09, 0.22)^1^	0.49 (0.20, 1.19)
Very Good (vs. poor)	0.30 (0.21, 0.41)^1^	0.24 (0.14, 0.42)^1^
Good (vs. poor)	0.41 (0.31, 0.54)^1^	0.33 (0.21, 0.50)^1^
Fair (vs. poor)	0.69 (0.52, 0.92)^2^	0.56 (0.39, 0.81)^3^

^1 ^p < 0.0001, ^2 ^p < 0.01, ^3 ^p < 0.05

note: Analyses of severe pain were limited to those with frequent pain.

Physician-driven participation was not significantly associated with the odds of frequent pain. However, elders with higher patient-driven participation had lower odds (OR, 0.82; 95% CI 0.75, 0.89) of frequent pain, confirming our hypothesis that persons who take a more active role in their medical treatment are less likely to experience pain. Individuals who had been diagnosed with arthritis at some point in their lives had a higher odds of frequent pain (OR, 3.62; 95% CI 3.03, 4.33) than those without arthritis. Those who rated their general health as excellent (OR, 0.14; 95% CI 0.09, 0.22), very good (OR, 0.30; 95% CI 0.21, 0.41), good (OR, 0.41; 95% CI 0.31, 0.54), and fair (OR, 0.69, CI 0.52, 0.92) had lower odds of frequent pain than those who rated their health as poor.

### Multivariate analyses of the odds of severe pain

Among those with frequent pain, there were no gender difference in the odds of severe pain. Persons of other race/ethnicity (primarily Black/African Americans) had a higher odds (OR, 2.28; 95% CI 1.24, 4.21) of severe pain than non-Hispanic whites. Income, marital status, rural residence, and frontier residence were not significantly associated with the odds of severe pain.

The number of physician visits was not significantly associated with severe pain. However, having insurance had a significant impact on the odds of severe pain. Compared to those without health insurance coverage, those with Medicare (OR, 0.41; 95% CI 0.19, 0.86), Medicaid (OR 0.35; 95% CI 0.17, 0.71), Medicare plus other private or government coverage (OR 0.31; 95% CI 0.15, 0.64), and those with other private or government coverage (OR 0.34; 95% CI 0.15, 0.76) had a significantly lower odds of severe pain. Although physician and patient-driven participation were not significantly related to the odds of severe pain, tenure with one's personal doctor was a significant factor. Elders who had been seeing their doctor for less than 1 year (OR, 0.51; 95% CI 0.27, 0.98), 3–4 years (OR, 0.56; 95% CI 0.33, 0.96), or 5 or more years (OR, 0.65; 95% CI 0.42, 0.99) had lower odds of severe pain than elders who had no personal doctor.

As expected, several health status measures were also significant. Those who had arthritis had higher odds of severe pain (OR, 1.54; 95% CI 1.01, 2.35) than those without arthritis. Finally, individuals who rated their general health as very good (OR, 0.24; 95% CI 0.14, 0.42), good (OR, 0.33; 95% CI 0.21, 0.50), and fair (OR, 0.56; 95% CI 0.39, 0.81) had lower odds of severe pain than those who rated their health as poor.

## Discussion

We found that patient-driven participation in decision-making was associated with lower odds of frequent pain, which is supported by previous research indicating that adult patients who are more actively engaged in their treatment have greater reductions in symptoms and improvement in health status [27], better psychological outcomes [28], and higher satisfaction with health care [29]. Thus, to delay or prevent the development of frequent pain, elderly patients may need to initiate discussions about symptoms with their physicians when they first experience them. However, because many elderly patients often defer to the doctor to initiate involvement in medical decisions [9-15], this may prove to be a difficult task. Little research has investigated how to promote active participation in medical care decision-making, but one prior study which involved sharing of a patient's medical record and the delivery of brief education about his/her disease prior to a physician visit demonstrated that patient involvement in decision making increased [30].

While neither physician nor patient-driven participation in decision making were significantly associated with pain severity, another factor related to the strength of the doctor-patient relationship was significant. In the present study, having a personal doctor, no matter how long the tenure of the relationship was, reduced the odds of severe pain. The finding of a beneficial effect of having a personal doctor, at least in terms of the severity of pain, is consistent with prior studies which have shown that having a usual source of care is positively correlated with an individual's access to the health care system [31-33], satisfaction with medical care [34], and promotion of proper medication use [35]. A usual, personal doctor undoubtedly has a more thorough knowledge of a patient's medical history and problems, which could enable him/her to more effectively manage pain treatment and coordinate care with specialists, if necessary. If this is the case, managers and leaders of physician clinics that have a high mix of elderly patients should ensure that patients can visit a regular doctor to promote better pain management.

In addition to having a personal doctor, access to any type of health insurance coverage was associated with the odds of severe pain. Approximately 12 percent of persons in the study sample reported that they had no health insurance coverage at all, including Medicare, and 21.5% of those without insurance had severe pain. The percentage of patients in this group with severe pain was nearly 2 times higher than the percentage of patients in the other insurance categories. Many older persons may not be eligible for public health insurance because they have not contributed to the social security system for a minimal amount of time. This may be particularly common in the southwestern United States where there are larger numbers of Hispanic immigrants. Expansion of health insurance coverage to this group could improve their ability to visit physicians and other health providers when they experience pain and, ultimately, lead to better pain management. Further research is warranted to more clearly elucidate how characteristics of different health insurance plans, such as gatekeeping and cost sharing, affect access to physician services for pain treatment.

Several demographic indicators were also significantly associated with frequent and severe pain. The gender differences beckon the question of whether medical care providers treat older women's pain less effectively or appropriately than men's. No differences were found between Hispanics and non-Hispanic whites, but other races (the majority of whom were Black/African American) had higher odds of severe pain than non-Hispanic whites. However, because of the heterogeneity of the other racial category, it is difficult to discern which particular racial groups experience severe pain. Research which includes greater numbers of other racial categories is thus warranted.

In regard to health status, the results support that individuals with three or more comorbid diseases have a relatively higher odds of frequent pain and severe pain than those with no comorbid diseases. One disease, arthritis, was of particular interest and therefore was treated as a separate variable. Almost two-thirds of the subjects had arthritis, which is not unexpected for persons age 65 and older. Persons with arthritis had a much higher odds (over 3 times the odds) odds of frequent pain than individuals without arthritis. Moreover, those with arthritis had approximately 1.5 times the odds of severe pain. The magnitudes of these associations imply that efforts to more effectively treat arthritis could lead to improvements in pain management.

While the present study has contributed to our understanding of the relationship between doctor-patient interactions and persistent pain, it is not without several limitations. Because the study was cross-sectional in design, it is impossible to infer any causal relationships. Although the pain measures were adapted from a nationally representative cohort study of older persons [23], they may not adequately reflect the frequency, duration, and severity of pain. The generalizability of the findings may be limited to regions of the southwestern United States that are similar in geographic and ethnic makeup, such as Texas, New Mexico, Colorado, Arizona, and California. However, we suspect that the associations found in the present study would hold true among elders residing in other parts of the United States. In summary, future studies should employ longitudinal designs, include more detailed measures of pain and be conducted among other populations.

## Conclusions

Despite these potential limitations, the present study suggests that several strategies could be implemented to limit the incidence and severity of pain among community-dwelling elders. Health policy makers and insurance companies might implement new reimbursement schemes to encourage visits to a personal physicians in order to improve pain and other health outcomes. Managers of physician clinics should consider organizing practices to ensure that older patients are able to make timely appointments with a personal provider. Finally, patients themselves could help reduce their chances of having frequent pain by becoming more involved in their care. These are just a few examples of how changes to the organization and delivery of care might affect pain-related health outcomes. Future research should evaluate how a range of physician characteristics (e.g. specialty and age), physician clinic characteristics (e.g. solo or group practice), insurance characteristics (e.g. HMO, PPO, or FFS coverage), and patient characteristics (e.g. trust in physician) influence pain and pain treatment.

## Competing interests

The author(s) declare that they have no competing interests.

## Authors' contributions

TFB conceived the overall study, performed the statistical analyses, and led the drafting of the manuscript. KTX assisted with the study design, interpretation of statistical findings, and drafting of the manuscript. JH assisted with interpretation of the findings and drafting of the clinical implications. GK contributed to data management, statistical analyses, and drafting of the methods section. All authors read and approved the manuscript.

## Pre-publication history

The pre-publication history for this paper can be accessed here:


